# Acute tryptophan depletion in healthy subjects increases preferences for negative reciprocity

**DOI:** 10.1371/journal.pone.0249339

**Published:** 2021-03-30

**Authors:** Paul Bengart, Theo Gruendler, Bodo Vogt

**Affiliations:** 1 Institute of Social Medicine and Health Systems Research, Otto von Guericke University Magdeburg, Magdeburg, Germany; 2 Department of Empirical Economics, Otto von Guericke University Magdeburg, Magdeburg, Germany; 3 Center for Military Mental Health, Military Hospital Berlin, Berlin, Germany; Heidelberg University, GERMANY

## Abstract

Reciprocity motivates to reward those who are kind (= positive reciprocity) and to punish those who are unkind (= negative reciprocity). The neurotransmitter serotonin (5-HT) modulates human behavior in numerous social situations, such as retaliation in response to perceived unfairness. In a placebo-controlled study, we used acute tryptophan depletion (ATD) to investigate the influence of available serotonin on choice behavior and reciprocity in the Hawk-Dove game. This game illustrates a conflict situation and incorporates two potential strategies: the cooperative Dove strategy and the uncooperative, more aggressive Hawk strategy. After strategic choices, we elicited the subjects’ expectations (= beliefs) regarding the opponent’s choices and controlled for risk preferences and current mood. We defined strategy choices as negative reciprocity when the participants opted for Hawk in response to an expected Hawk. We hypothesized that the ATD-induced reduction of 5-HT availability would increase participants’ preferences for negative reciprocity. Generalized estimating equations reveal no significant main effect of ATD on assessed belief, mood, or risk attitude. But assessment of ATD’s marginal effects over beliefs suggests that ATD significantly increases the tendency for negative reciprocity, whereas positive reciprocity (Dove in response to an expected Dove) is unaffected. We could therefore demonstrate that 5-HT availability mediates (negative) reciprocal behavior in social decision-making.

## Introduction

People’s decisions often involve a conflict between self-interest and the interests of others. In these social dilemmas, traditional economic models often assume that people act purely selfishly, meaning that they maximize their own material or monetary outcomes regardless of the consequences for others [1, 2]. There is however ample evidence that contradicts these assumptions and that shows that people do not only care about their own outcomes, but also those of others [3–5]. Likewise, people shape their decisions according to others’ behavior and/or the perceived intentions behind it, which can lead to reciprocal behavior [6, 7]. Reciprocity is reflected by the decision-maker’s willingness to reward those who are perceived as kind (positive reciprocity) and punish those who are perceived as unkind (negative reciprocity), “even if this is costly and provides neither present nor future material rewards for the reciprocator” [8, p. 3].

Of course, acting (un)kindly toward others does not guarantee that others will reciprocate this (un)kindness. People may perceive the same external events (e.g. others’ behavior) in the same decision-making context differently—what one person perceives as kind, another may perceive as unkind [9]. Even if perceptions are similar, different patterns of behavior may occur [10]—whereas one person might react to perceived unfairness with costly punishment (negative reciprocity), another person might tolerate it to achieve self-interested goals (e.g., material gain). Such variation in responses to others’ behavior can also occur at the intrapersonal level when deciding differently at different times, even if the decision-making context and the external events (e.g. others’ behavior) remain unchanged [11]. The factors that contribute to these fluctuations are however not well-understood.

Serotonin (5-HT) has been implicated in modulating a broad range of cognitive functions [12, 13], in having an important role in behavioral inhibition and in processing aversive events in a social context [2, 14, 15]. In the face of expected punishment, a reduction of 5-HT levels leads to a disinhibition of behavior [16] and this also applies to internal adverse events [17]. Acute L-tryptophan depletion (ATD) is a dietary method that is considered safe and effective and that has been widely used in social, behavioral, and economic sciences (e.g., [18–20]). It is based on administering a tryptophan-free amino acid drink that depletes blood plasma tryptophan, an amino acid that is required for serotonin synthesis in the human brain (more details in the method section) [21, 22].

Using the ATD technique, Wood and colleagues [23] examined whether reducing subjects’ brain serotonin levels had an impact on cooperative behavior in the Prisoner’s Dilemma (PD) and demonstrated that ATD decreased the emergence of cooperative strategies. This observation was interpreted as that the induced serotonin depletion led to a decrease in reciprocal altruism, which, as the authors state, is a driving force for cooperation in the PD. Using the same experimental manipulation of 5-HT availability, Crockett and colleagues [24] found that those who underwent ATD in the Ultimatum Game (UG) rejected unfair offers more frequently than those in the placebo group. This rejection rate of unfair offers can also be interpreted as an elevation of negative reciprocity [25].

Despite convincing experimental support for the role of ATD in reciprocity, several unresolved issues remain. First, because a person’s behavior may differ from one type of social dilemma to another, it is not clear whether the findings from the abovementioned studies are limited to the specific social dilemma types the PD and UG represent [26, 27]. Moreover, as a recent study suggested, it is questionable whether the UG is an appropriate experimental paradigm to measure individuals’ disposition toward altruistic punishment behavior or reciprocity [28]. In a series of experiments, the authors found that neither the behavior in other social preference games nor the negative reciprocity self-report scale were significantly correlated with the rejection rate of unfair offers. Therefore, further investigations using other game theoretic approaches are needed for a more complete understanding of the relationship between ATD and reciprocity. Second, as Siegel and Crockett [25] noted, it is not known “whether serotonin modulates social preferences themselves, or alternatively, the beliefs upon which the preferences are predicated” (p. 45). With that statement, they refer to decision-makers’ sensitivity to their own beliefs about others’ actions in strategic interactions—a factor that previous studies on 5-HT neglected.

This study was set up to address the abovementioned issues. We conducted a double-blind, placebo-controlled, randomized experiment based on the Hawk-Dove game in conjunction with the ATD technique. We used the two-person Hawk-Dove game, as this “is perhaps the ideal game for contrasting fairness and self-interested preferences” [29, p. 171]. The Hawk-Dove game represents a conflict situation with two possible strategies: the uncooperative and potentially aggressive Hawk strategy (i.e., defection) and the cooperative Dove strategy [30, 31]. In addition to the strategy choices, we elicited subjects’ beliefs regarding an opponent’s actions. This allowed us to determine whether and how ATD influenced choice behavior and to distinguish between self-interested (i.e., payoff maximization) and fairness-related (i.e., reciprocity) preferences underlying the observed choices. Furthermore, we controlled for demographics, general risk preferences, and mood.

We hypothesized that the ATD group would respond to the expected Hawk with Hawk more often than the placebo group. We therefore expected participants with reduced available 5-HT to display negative reciprocal behavior more frequently.

## Materials and method

### Participants and procedure

We recruited 49 healthy participants at the Otto von Guericke University Magdeburg to participate in the between-subjects experiment. This sample size is within the range of other studies using the ATD technique [16, 18, 24] but does not allow a strong inference due to the limited sample size. Due to interactions with the female estrogen cycle [32, 33], only males were invited. The study was conducted in accordance with ethical standards for human research and under the terms and conditions of the MaXLab, the Magdeburg Experimental Laboratory of Economic Research. The board of the MaXLab, which granted the ethical approval for this study, consisted of senior faculty members of the Faculty of Economics and Management at the Otto von Guericke University Magdeburg. All participants signed a consent form before they took part in the experiment. We used the following criteria to exclude participants: self-reported neurological, psychiatric, cardiac, renal disorder, drug abuse, and serotonergic conditions.

**Fig 1:**
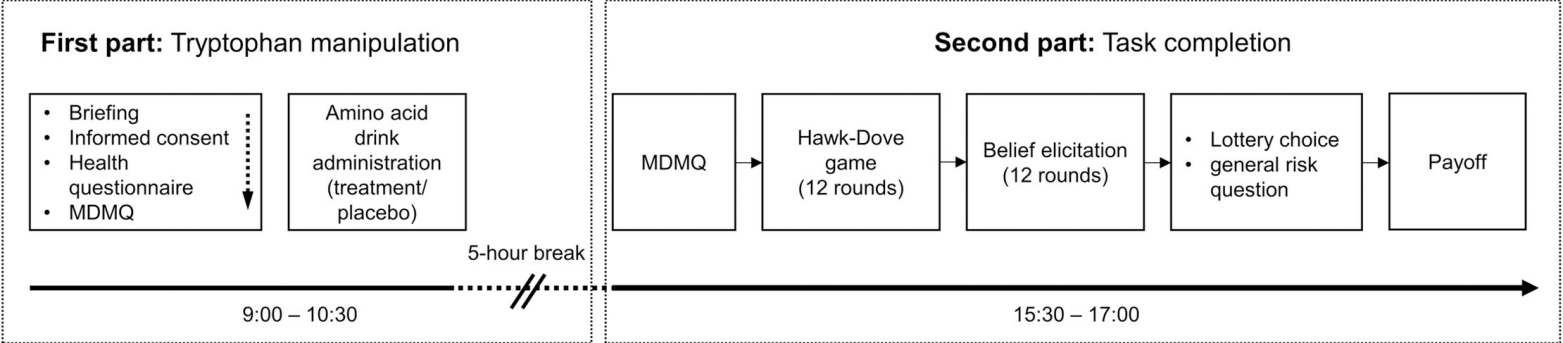
Experimental procedure. MDMQ = multidimensional mood state questionnaire.

The experiment was conducted in two time slots on the same day (Fig 1). The first slot began at 09:00 and ended at 10:30. Participants entered individual cubicles to fill out the self-report exclusion screening questionnaire and the (pre-treatment) multidimensional mood state questionnaire (MDMQ) [34]. This measurement was included due to ATD’s reported effects on mood [35]. The elicitation was repeated in the second part of the experiment, which enabled pre-treatment and post-treatment mood comparison. In a randomized double-blind fashion, either a tryptophan depleting drink (ATD group; *N* = 25) or a placebo drink (placebo group; *N* = 24) was administered to each participant. Both drinks had similar taste and consisted of an amino acid mixture. The ATD group’s drink contained the same ingredients as the placebo group’s drink, except for the absence of tryptophan leading to reduced serotonin synthesis [36, 37]:

- ATD group’s drink: *Water (200 ml)*, *L-Alanin (5*.*5 g)*, *L-Arginin (4*.*9 g)*, *L-Cystein (2*.*7 g)*, *Glycin (3*.*2 g)*, *L-Histidin (3*.*2 g)*, *L-Isoleucin (8*.*0 g)*, *L-Leucin (13*.*5 g)*, *L-Lysin Monohydrochlorid (8*.*9 g)*, *L-Methionin (3*.*0 g)*, *L-Phenylalanin (5*.*7 g)*, *L-Prolin (12*.*2 g)*, *L-Serin (6*.*9 g)*, *L-Threonin (6*.*5 g)*, *L-Tyrosin (6*.*9 g)*, *and L-Valin (8*.*9 g)*.
- Placebo group’s drink: *Same ingredients as the ATD group’s drink*, *plus 2*.*3 g L-Tryptophan*.

To allow for tryptophan clearance, the second part of the experiment commenced after five hours.

The second part started with the (post-treatment) MDMQ, followed by written instructions for the (1) Hawk-Dove game task and the (2) belief elicitation task. Twelve versions of the Hawk-Dove game were given. After all games were played, participants were asked to report their beliefs about others’ actions. Only one game became relevant for payoff. Afterwards, the participants were instructed to perform the incentivized (3) lottery choice task and to answer the *general risk question* [38].

To avoid learning or reputation effects, no feedback about the opponent’s strategy choice was provided. After all tasks were completed, participants were randomly pairwise matched to determine their payoff.

### Experimental tasks

#### Hawk-Dove game

**Fig 2:**
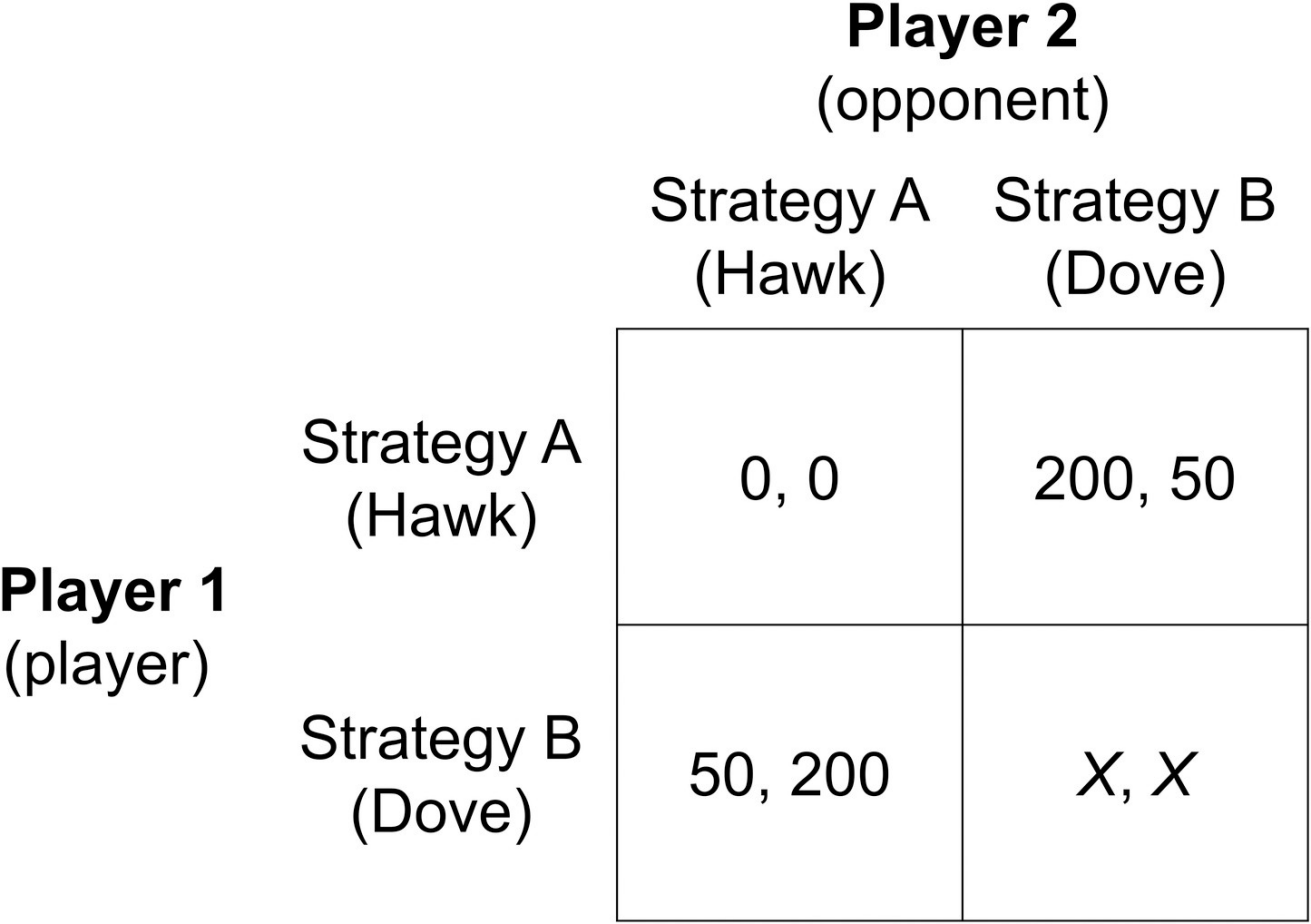
The Hawk-Dove game’s payoff structure with incrementally increasing incentives for mutual cooperation. *X* = {60, 70, 80, 90, 100, 110, 120, 130, 140, 150, 160, 170}. The number before each comma is the player’s payoff and the number after each comma is the opponent’s payoff. The exchange rate is €0.05 for each point earned. Only one game is payoff-relevant.

Participants played the Hawk-Dove game with a symmetric payoff matrix by choosing either Hawk (Strategy A) or Dove (Strategy B). Fig 2 shows the payoffs in points (1 point = €0.05) to each player for every possible strategy pair:

The row player (referred to as “player”) and the column player (referred to as “opponent”) face the same payoff function. Each player’s payoff depends on his own choice and that of the opponent—when the opponent chose Hawk, the player would receive a payoff of 50 when choosing Dove and a payoff of 0 when also choosing Hawk. Choosing Dove in response to Hawk (Dove/Hawk) therefore yielded a higher payoff than choosing Hawk in response to Hawk (Hawk/Hawk). Alternatively, if the opponent chose Dove, a player would receive a payoff of 200 if he chose Hawk (Hawk/Dove) and a payoff of *X* if he also chose Dove (Dove/Dove). Payoff maximization was therefore achieved by using a strategy that differed from that of the opponent. A strategy that maximizes the individual payoff given another’s action is also known as the best response (strategy). Of course, the opponent’s choice is not open and has to be inferred by the player.

We manipulated the incentive level for the constellation Dove/Dove, which can be termed as mutual cooperation [39], by incrementally increasing its values by 10 points (*X* = 60 *to X* = 170). All the participants played each of the 12 versions of the Hawk-Dove game. This approach enabled us to evaluate the explicit incentive for mutual cooperation’s effects on strategy choices and beliefs. In addition to the between-groups comparison, our experimental design therefore made it possible to investigate changes in responses (i.e., strategy choices and beliefs) within each subject.

#### Belief elicitation

As our study contained no information about the opponent’s strategy choice, the term “best response” refers to choosing the option with the highest expected payoff, given the beliefs about the other’s action [4]. This means that a payoff-maximizing player’s best response was to choose Dove (Hawk) if he expected the opponent to choose Hawk (Dove). After the participants completed all versions of the Hawk-Dove game, we asked them to state which strategy they believed the opponent had chosen and then to state how confident they were about their guess on a scale from 0 (certain Dove) to 100 (certain Hawk). To encourage participants to reveal their actual probabilistic beliefs, this task was designed to be incentive compatible using Nyarko and Schotter’s [40] quadratic scoring rule (QSR). The payoff *S* of each subject and each estimation *i* is calculated as follows:

If the strategy choice prediction was correct:

$$S_{i}=200*\left[1-{\left(1-\frac{p_{i}}{100}\right)}^{2}\right]$$ (1)

If the strategy choice prediction was not correct:

$$S_{i}=200*\left[1-{\left(\frac{p_{i}}{100}\right)}^{2}\right]$$ (2)

As the formulas above show, the number of points *S* (1 point = €0.05) that a player could earn, depended on whether the player’s prediction matched the opponent’s actual strategy choice and on his stated probabilistic belief *p_i_* (hereafter simply referred to as “belief”) he assigned to his guess. Similar to the Hawk-Dove task, only one decision *i* was randomly selected that became payoff relevant to the participants. To facilitate a better understanding of the QSR and therefore the payoff structure of the belief elicitation task, we provided participants with a table showing the possible payoffs as a function of the stated beliefs and of the opponent’s actual strategy choice. The instructions for the belief elicitation task and a sample of the answer sheet can be found in the S1 Appendix.

As stated, individuals do not necessarily act as payoff-maximizing agents. Instead, they are often willing to sacrifice their own payoff by not playing the best response, either to reward an opponent’s kind behavior (= positive reciprocity) or to punish unkind behavior [41]. Mere expectations about others’ behavior and associated beliefs about others’ motives are sufficient to evoke reward or punishment behavior in the decision-maker [42, 43]. For instance, if a player expects that his opponent chose Hawk, he may perceive the opponent’s intentions as selfish or unkind. This thinking can motivate the player to punish the opponent by also choosing Hawk instead of the best response, which is Dove. In other words, the player sacrifices his own expected payoff to reduce that of the opponent. Besides the opponent’s intentions, inequity aversion may also motivate reciprocal behavior [44]. The characteristics of an inequity-averse individual entails deriving disutility from an inequitable payoff distribution between self and others, regardless of others’ intentions. In the Hawk-Dove game, inequity aversion may motivate the player to choose the same strategy as the opponent, which would yield equal payoffs for both. Our study, however, was not designed to distinguish between different motives that may lead to reciprocal behavior. Instead, it distinguishes between preferences for payoff maximization and preferences for reciprocity, as described in Table 1:

**Table 1:** Preferences inferred from strategy choices and beliefs.

Strategy-belief combination	Player’s stated belief	Player’s strategy choice	Player’s expected payoff	Opponent’s expected payoff	Inferred preference
1	Dove	Hawk	200	50	Payoff maximization
2	Dove	Dove	*X*	*X*	Positive reciprocity
3	Hawk	Dove	50	200	Payoff maximization
4	Hawk	Hawk	0	0	Negative reciprocity

The preferences were inferred from the combination of the player’s belief and his strategy choice. *X* = {60, 70, 80, 90, 100, 110, 120, 130, 140, 150, 160, 170}.

#### Risk attitude assessment

As evidence suggests that risk attitudes affect strategy choices in the Hawk-Dove game [45], we wanted to rule out the possibility that potential differences in risk attitudes could be responsible for potential between-group differences in strategy choices. To this end, we elicited participants’ risk attitudes using a lottery choice task adapted from Holt and Laury [46]. This task is comprised of 11 lottery pairs, each of which included a risky lottery (Lottery A) and a less risky one (Lottery B) (see S1 Table).

We asked participants to choose between each of the presented lottery pairs and informed them that their decisions would be payoff-relevant. The risky lottery offered a chance *p* to win 200 points and a chance of 1-*p* to win nothing. The less risky lottery, on the other hand, yielded 120 points (*p*) or 50 points (1-*p*), respectively. The chance *p* to win the higher amount was 100% (1-*p* = 0%) in the first lottery pair. With each subsequent lottery pair, *p* was incrementally reduced by 10%, with the consequence that the expected value of Lottery A decreased while the expected value of Lottery B increased. To assess the participant’s risk attitude, we considered the number of the lottery pair where he switched from choosing Lottery A to choosing Lottery B. The later the participant switched, the more risk-seeking he was considered to be.

In contrast to tasks 1 and 2, this task had no social component. The payoff only depended on participants’ choices and on the outcome of the two-stage random draw that we conducted at the end of the experiment.

### Behavioral analysis

In the first analysis, we considered the participants’ responses (1) across all 12 versions of the Hawk-Dove game and (2) the related belief elicitation task. To overcome the problem of non-independent responses from the same participant, we applied generalized estimating equations (GEE) [47], an analytical tool that has been widely used in other studies that investigated the role of brain chemicals on human behavior in strategic interaction [19, 48, 49]. It takes possible within-subject correlations into account and has been suggested to yield more precise estimates than its alternatives, the mixed-effects approach and the repeated measures ANOVA, when only smaller sample size is available [50].

In the first model (Model 1), we examined the main effects of ATD (0 = placebo, 1 = ATD), the stated beliefs (transformed into the unit interval [0,1]), the incentives for mutual cooperation (incentive DD), and the lottery task’s switching points (= risk attitude) on the strategy choices (0 = Dove, 1 = Hawk). We treated the variables incentive DD and risk attitude as continuous variables. We excluded data obtained from two participants from this and following models, because these participants switched more than once between lotteries in the lottery choice task. We could therefore not determine their risk attitudes.

In a second analysis, we tested the ATD’s moderating effect on the relationship between beliefs and strategic choices. Following Baron and Kenny [51], a significant effect of the interaction term, while all variables used in the interaction term are controlled, indicates a moderator effect. Model 2, therefore, included the interaction term ‘ATD × belief’ and controlled for the main effects of the predictors ATD and beliefs. Further control variables were incentive DD and risk attitude.

We also performed a post hoc power analysis using Monte Carlo simulations [52]. As a robustness check, we repeated the analysis using a GEE linear regression and a mixed-effects logistic regression (see S2 Table for full results). In addition, we conducted a randomization inference (RI) test with 2,000 permutations [53]. It is a more conservative test that has the advantage of being insensitive to the sample size [54].

To further examine this effect of negative reciprocity, we conducted another GEE analysis (Model 3) with negative reciprocity as the dependent variable that indicated whether a participant behaved in a negative reciprocal manner (1) or not (0). To this end, we calculated for each version *X* of the Hawk-Dove game the probabilities *p*_*x*_*(H)* of encountering a Hawk at which the expected payoff for choosing Hawk was the same as the payoff for choosing Dove: *p*_*60*_*(H)* = .737, *p*_*70*_*(H)* = .722, *p*_*80*_*(H)* = .706, *p*_*90*_*(H)* = .688, *p*_*100*_*(H)* = .667, *p*_*110*_*(H)* = .643, *p*_*120*_*(H)* = .615, *p*_*130*_*(H)* = .583, *p*_*140*_*(H)* = .545, *p*_*150*_*(H)* = .500, *p*_*160*_*(H)* = .444, *p*_*170*_*(H)* = .375. This implied that if a payoff-maximizing player’s belief was equal to the value of *p*_*x*_*(H)*, they should have been indifferent toward choosing either Hawk or Dove. Second, based on the actual strategy choices and the comparison between the stated beliefs and *p*_*x*_*(H)*, participants’ responses were classified into three categories:

1. Best response: Hawk choice given belief < *p*_*x*_*(H)* or Dove choice given belief > *p*_*x*_*(H)*
2. Negative reciprocity: Hawk choice given belief > *p*_*x*_*(H)*
3. Positive reciprocity: Dove choice given belief < *p*_*x*_*(H)*

In a third step, we coded the dependent variable negative reciprocity as 1 if the response fell into category 2 and 0 if the response fell into either category 1 or 3. The predictors in the present model are ATD (0 = placebo, 1 = ATD), incentive DD (continuous), and risk attitude (continuous). Belief was not included as a predictor variable, as it is a component of the dependent variable negative reciprocity. We present the results of the corresponding model estimation in Table 4.

Lastly, we analyzed whether ATD influenced participants’ beliefs (Model 4). To this end, we performed a linear GEE with beliefs [0, 1] as the dependent variable and ATD, incentive DD, and risk attitudes as the predictors.

## Results

### Preliminary analysis

Comparing the ATD group with the placebo group, we found no significant differences in age (*M*_ATD_ = 24.56, SD_ATD_ = .54 vs. *M*_placebo_ = 25.04, SD_placebo_ = .60, *t(47)* = .55, *p* = .554), height (*M*_ATD_ = 181.96, SD_ATD_ = 1.28 vs. *M*_placebo_ = 184.79, SD_placebo_ = 1.60, *t(47)* = 1.39, *p* = .171), weight (*M*_ATD_ = 81.00, SD_ATD_ = 2.31 vs. *M*_placebo_ = 85.33, SD_placebo_ = 2.50, *t(47)* = 1.42, *p* = .162), and monthly income (*M*_ATD_ = 603.75, SD_ATD_ = 205.70 vs. *M*_placebo_ = 674.58, SD_placebo_ = 266.88, *t(47)* = 1.03, *p* = .308). Furthermore, no differences in risk attitudes (*t*(45) = -.50, p = .621) [46] and self-assessed general risk attitudes were present (*t*(47) = .83, *p* >.410) [38]. The ANOVA on the mean scores of the post-treatment MDMQ revealed that there were no significant group differences in pleasantness (*F*(1;48) = 2.06, *p* = .158), wakefulness (*F*(1;48) = 1.56, *p* = .217), and calmness (*F*(1;48) = .40, p = .529). There were also no significant differences between the pre-treatment and post-treatment MDMQ scores (pleasantness (*F*(1;48) = .26, *p* = .615), wakefulness (*F*(1;48) = .07, *p* = .786), and calmness (*F*(1;48) = 2.05, *p* = .159)).

### Determinants of strategy choices

The results from Model 1 in Table 2 indicate a positive effect of the probabilistic beliefs that the opponent will choose Hawk on the likelihood of selecting the Hawk strategy (*z* = 3.68, p < .001). The results also show that the effect of ATD on the choice likelihood of a Hawk strategy is only significant at the 10% level (*z* = 1.65, *p* = .099). We found that an increase of the incentive for mutual cooperation (incentive DD) significantly decreased the likelihood of selecting Hawk (*z* = -7.64, *p* < .001), whereas the risk attitude had no significant impact on the strategy choices (*z* = .53, *p* = .529).

**Table 2:** Results of th*e* generalized estimating equations (GEE) logistic regression predicting strategy choices.

	Model 1			
	DV: Strategy choice (0 = Dove, 1 = Hawk)			
	Coef. (OR).	RSE	z-value	p-value
ATD	.68 (1.97)	.81	1.65	.099
Belief	1.38 (3.98)	1.49	3.68	< .001
Incentive DD	-.02 (0.98)	.00	-7.64	< .001
Risk attitude	.07 (1.08)	1.12	.53	.529
Constant	-.47 (1.59)	1.31	-.57	.570
N =	47*12			
Wald χ^2^	110.69			< .001

All p-values are two-sided.

Coef. = Coefficient, OR = Odds ratio, RSE = Robust standard error, Incentive DD = Incentive for mutual cooperation (Dove, Dove).

**Fig 3:**
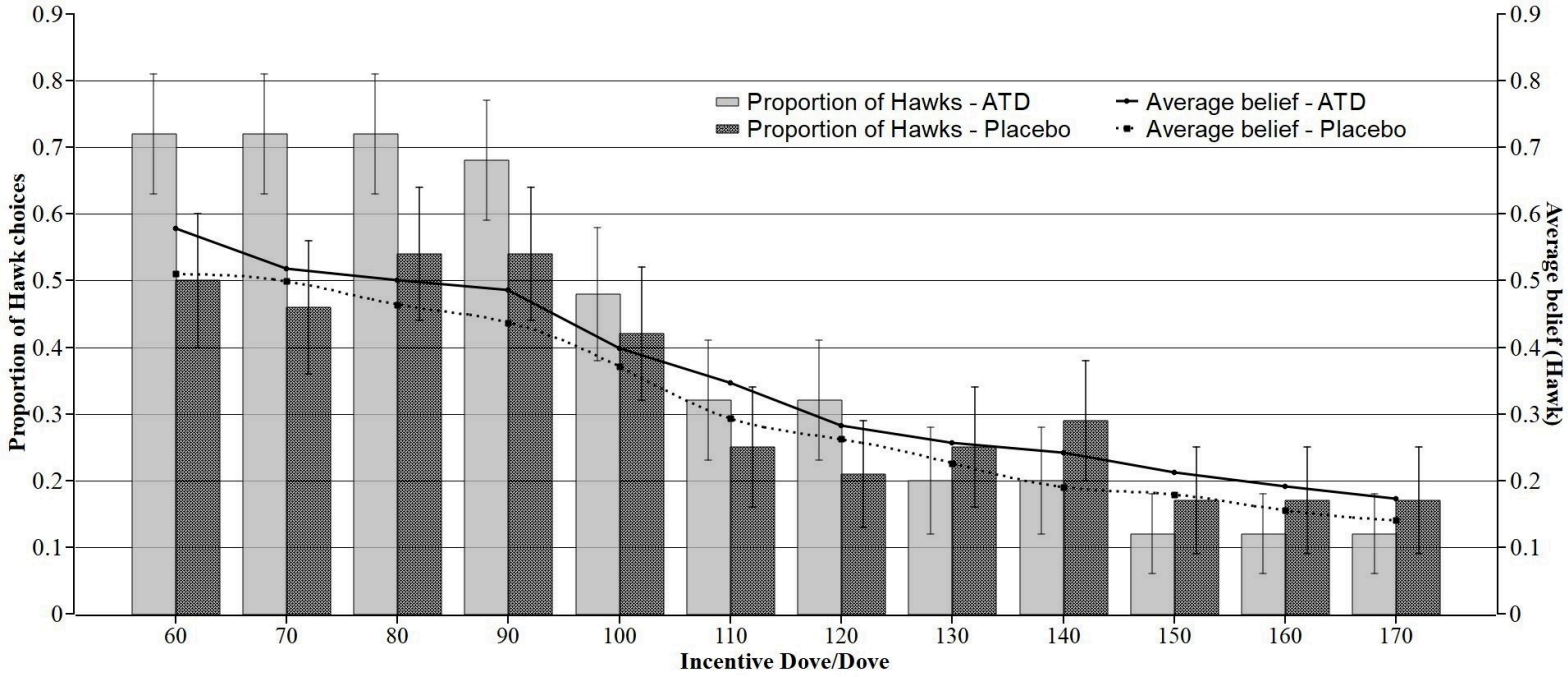
Proportion of Hawk choices (bars) and average beliefs that the opponent will play Hawk (Strategy A) as a function of the incentive for mutual cooperation (incentive DD). Error bars represent the standard error. When the belief for Hawk is high, the choice share of Hawk is also high, and vice versa. The higher the incentive for mutual cooperation (Dove-Dove), the less often the participants chose Hawk (raw data in the S3 Table).

The results suggest that participants behaved reciprocally in accordance with their own beliefs and did not engage in payoff-maximizing behavior. The participants’ tendency for reciprocal behavior is also clearly visible in Fig 3.

### Serotonin effects on strategy choices

We found that ATD had a moderating effect on the relationship between beliefs and strategic choices (Model 2) (*z* = 4.52, *p* < .001) and that the incentive for mutual cooperation had a strong negative effect on choosing Hawk (*z* = -8.25, *p* < .001), whereas risk attitude had no effect (Table 3). These results are robust in estimating the model using a GEE linear regression as well as using a mixed-effects logistic regression (see S2 Table). However, using the randomization inference test confirms the robustness of the interaction effect of ATD × Belief only when excluding decisions that participants made in the Hawk-Dove game with incentive DD = 160 and incentive DD = 170 (over decisions with incentive DD ≤ 150: *RI-p =* .*043*, *SE =* .*004*; over all decisions: *RI-p =* .061, *SE =* .005).

**Table 3:** Results of the generalized estimating equations (GEE) logistic regression testing the interaction effect of treatment and beliefs on strategy choices.

	Model 2			
	DV: Strategy choice (0 = Dove, 1 = Hawk)			
	Coef. (OR)	RSE	z-value	p-value
ATD	-.87 (.42)	.54	-1.60	.109
Belief	-.20 (.82)	.49	-.40	.687
ATD × Belief	3.37 (29.18)	.75	4.52	< .001
Incentive DD	-0.02 (.98)	.00	-8.25	< .001
Risk attitude	0.20 (1.21)	.13	1.55	.120
Constant	0.62 (1.85)	.82	.75	.455
N =	47*12			
Wald χ^2^	121.75			< .001

All p-values are two-sided.

Coef. = Coefficient, OR = Odds ratio, RSE = Robust standard error, Incentive DD = Incentive for mutual cooperation (Dove, Dove).

The post hoc power to detect the ATD × belief interaction effect revealed a statistical power of .92 when the logit link for the dependent variable (strategy choice) is used and .60 when the dependent variable is treated as a linear variable (see S2 Table). However, post hoc power analyses are susceptible to bias and their results should be treated with caution [55].

**Fig 4:**
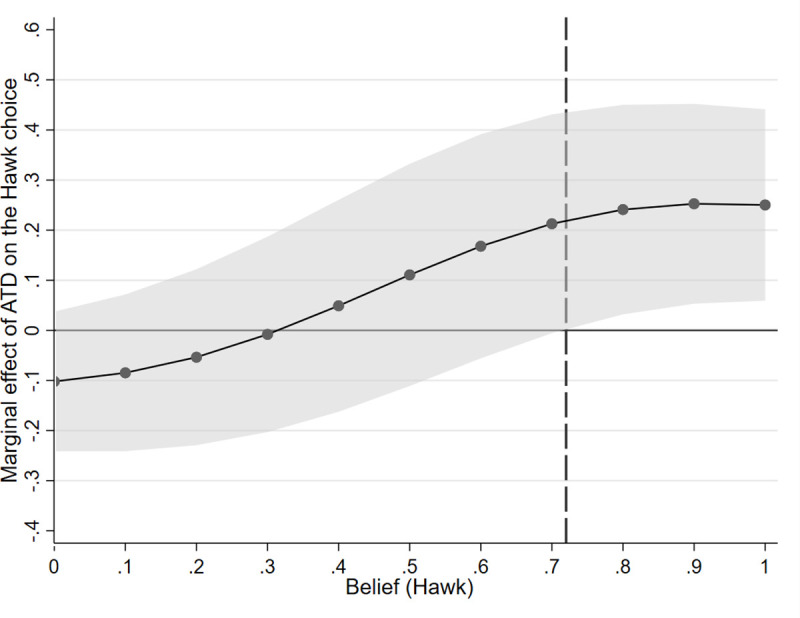
Marginal effects of ATD with a 95% confidence interval as a function of beliefs. The vertical dot line represents a belief value beyond which the probability of a Hawk choice differs significantly between the ATD group and the placebo group (p < .05, two-tailed).

To investigate the interaction effect further and facilitate its interpretation, we calculated ATD’s marginal effects across all levels of beliefs. Marginal effects can be understood as predicted changes in a probability of success (= choosing Hawk) when the categorial variable changes from 0 (= placebo) to 1 (= ATD) [56]. The results are presented in Fig 4.

The ATD and placebo groups differed significantly (*p* < .05) from each other in strategy choices only when Hawk was expected with a high probability of ≥ .72, but not when Dove was expected. This means that participants who received ATD were more likely than non-depleted participants to respond to a believed Hawk with a Hawk choice. The results obtained for Model 3 (Table 4) with negative reciprocity as the dependent variable, which show a significant influence of ATP (*z* = 3.11, *p* = .002) and a negative impact of the incentive for mutual cooperation (*z* = -6.41, *p* < .001), also support this relationship.

**Table 4:** Generalized estimating equations (GEE) logistic regression predicting negative reciprocity.

	Model 3			
	DV: Negative reciprocity (0 = no, 1 = yes)			
	Coef. (OR)	RSE	z-value	p-value
ATD	1.54 (4.65)	.49	3.11	.002
Incentive DD	-.03 (0.81)	.00	-6.41	< .001
Risk attitude	-.21 (1.05)	.14	-1.48	.139
Constant	1.35 (3.84)	.89	1.51	.130
N =	47*12			
Wald χ^2^	50.50			< .001

All p-values are two-sided.

Coef. = Coefficient, OR = Odds ratio, RSE = Robust standard error, Incentive DD = Incentive for mutual cooperation (Dove, Dove).

In conclusion, Model 1 supports the idea of reciprocal behavior and not payoff maximization. The results from Model 2, Model 3, and the marginal effect analysis further support the hypothesis of enhanced negative reciprocity under ATD.

### Serotonin effects on beliefs

To ascertain whether beliefs mediated the differences in strategy choices between the ATD group and the placebo group, we conducted a GEE linear regression model with belief [0,1] as the dependent variable (Table 5, Model 4). We defined the treatment group, incentive DD, and risk attitude as independent variables. As is apparent in Fig 3, the differences between the ATD group and the placebo group are minimal at every incentive level for mutual cooperation. The results of our model confirm the presumption that these differences are not statistically significant. Neither the coefficient of the variable ATD (*Coef*. = .035; *p* = .605) nor the coefficient of the risk attitude is statistically significant (*Coef*. = .027; *p* = .17). Only the coefficient of the incentive DD (*Coef*. = -.004, *p* < .001) influences the beliefs significantly.

**Table 5:** Generalized estimating equations (GEE) linear regression model predicting beliefs.

	Model 4			
	DV: Beliefs [0, 1]			
	Coef.	RSE	z-value	p-value
ATD	.035	.068	.52	.605
Incentive DD	-.004	.019	-16.34	< .001
Risk attitude	.027	.019	1.39	.165
Constant	.58	.127	4.57	< .001
N =	47*12			
Wald χ^2^	269.38			< .001

All p-values are two-sided.

Coef. = Coefficient, RSE = Robust standard error, Incentive DD = Incentive for mutual cooperation (Dove, Dove).

## Discussion

We examined the effect of reduced availability of serotonin on reciprocity in a competitive social context represented by the Hawk-Dove game. We hypothesized that lowering the available serotonin increases the tendency for negative reciprocity. To test this hypothesis, we conducted an experiment using a placebo-controlled between-subjects design in which the participants were administrated an amino acid mixture either lacking L-tryptophan (ATD group), a necessary precursor of serotonin synthesis, or containing L-tryptophan (placebo group). Thereafter, the participants played 12 versions of the Hawk-Dove game, differing in the incentive for mutual cooperation (with a payoff when both players chose Dove). In addition to the strategy choices, we elicited the participants’ beliefs about the other’s action and controlled for risk attitudes and mood. To the best of our knowledge, this is the first study that investigates whether the ATD’s impact in social decision-making is due to its potential influence on the beliefs.

We observed a significant interaction between ATD and expressed beliefs, which suggests that the participants who received ATD were more likely to behave in a reciprocal manner and were therefore less likely to behave in a payoff-maximizing manner than those who received placebo. A closer examination of this interaction revealed that ATD affected only negative reciprocal behavior, meaning choosing Hawk in response to Hawk, but not the positive, which is Dove in response to Dove. In contrast to Wood and colleagues [23], we found no (negative) effect of ATD on cooperative strategy choices, but noted a slight trend toward less cooperative strategy choices in the ATD group (*p* = .099). There was no effect of ATD on beliefs, risk attitudes, and mood. In line with Rubinstein and Salant [57], we found that across the two experimental groups participants’ strategy choices were driven by their beliefs—they tended to choose the same strategy they believed their opponent would choose. The extent of the incentive for mutual cooperation also significantly influenced participants’ strategy choices as well as their beliefs. The participants’ risk attitudes, on the other hand, did not play any role.

One of the driving forces for reciprocal behavior is inequity aversion (IA) that can be divided into the advantageous IA (receiving more than others) and disadvantageous IA (receiving less than others), whereby the latter IA proves to be more prevalent [44]. As Gao and colleagues [58] demonstrated, these two types of IA differ in their underlying neurocognitive processes: The advantageous IA involves social processing and mentalizing, whereas disadvantageous IA involves emotional and conflict processing. ATD is suggested to impair emotional self-regulation, resulting in an increase in emotional-driven behavior that, in turn, is associated with economically unfavorable decisions [for a review, see 16, 59]. The Hawk-Dove game’s payoff structure (Fig 2) shows that disadvantageous outcomes can be prevented by choosing Hawk. An increase in disadvantageous IA in the participants who underwent ATD may also explain why ATD increased the likelihood of negative reciprocity. The advantageous IA might play a less important role in the competitive Hawk-Dove game, since its effect diminishes when decisions are made in a competitive context [60].

Another potential explanation derives from the characterization of negative reciprocity as willingness to harm others [61]. Harm aversion prevents subjects from taking actions that are harmful to others [62, 63]. Its extent may depend on 5-HT, whose increase has been reported to enhance harm aversion [49]. This inhibitory effect of 5-HT on harm aversion could also explain the ATD-induced increase in negative reciprocity. In this way our results support the idea of a link between 5-HT and harm aversion. Further, as Rogers and colleagues [64] have described, ATD appears to alter reward processing in that ATD led to a reduction in discrimination between smaller and larger rewards. Considering that choosing Dove in response to Hawk yielded the third smallest payoff in our experiment, the participants who received ATD might be more indifferent toward choosing between the small payoff and the zero payoff. Consequently, ATD might shift the focus from pursuing monetary self-interest to pursuing non-monetary interests. However, because Faulkner [65] failed to replicate the findings of Rogers and colleagues [64], this explanation should be taken cautiously.

A limitation of this study is our restriction of ATD’s effects in males. The effects of ATD may affect females differently (e.g., free tryptophan levels and mood levels) [66], therefore we cannot rule out the possibility that the effect of ATD on beliefs and strategy choices may differ between genders. Furthermore, although our sample size is fairly similar to or larger than in other studies that used the ATD technique [16, 18, 24], future studies with larger sample sizes are needed to verify our results. Another limitation is that we only examined the effects of reduced 5-HT availability. We can therefore not state whether higher 5-HT levels will lower negative reciprocity in the Hawk-Dove game. Furthermore, because our experiment is a between-subjects design, the participants’ unobserved background characteristics, such as a predisposition toward reciprocal behavior, might have biased our results on group differences. Moreover, our study design does not allow a distinction between different motives that led to reciprocal behavior or determining which exact personal states that could be responsible for the differences in reciprocal behavior between the two experimental groups were affected by ATD. It is also not clear whether our results can be generalized to another decision-making context. Future research is therefore needed, for example, to evaluate whether ATD has an effect on individuals’ beliefs in less competitive environments. Another possible limitation concerns the validity of stated beliefs. There are studies suggesting that individuals do not necessarily reveal their true beliefs about others’ behavior when their own actions are selfish or negatively affect the others [67, 68]. According to these studies, individuals tend to distort their beliefs in order to justify their actions to themselves and to not reveal their beliefs truthfully in order to make their behavior appear socially more acceptable to others (e.g., experimenter). On the other hand, a recent study [69], which was a replication and extension of [68], failed to provide evidence for self-serving belief manipulation. Although we cannot rule out that the Hawk player might have stated higher than their true (probabilistic) beliefs that the opponent would play Hawk, we are confident that this potential bias had little effect on the findings regarding the effect of ATD on negative reciprocity, as the beliefs were unaffected by ATD (Table 5).

## Supporting information

S1 Appendix Experimental instruction for the belief elicitation task. (DOCX)

S1 Table Lottery choice task. (DOCX)

S2 Table Robustness checks on the GEE regression testing the interaction effect of treatment and beliefs on strategy choices (i.e., Model 2). (DOCX)

S3 Table Strategy choices and beliefs over the incentive levels for mutual cooperation. (DOCX)

S1 Data (XLSX)
