# Supplementary material for: Acute tryptophan depletion in healthy subjects increases preferences for negative reciprocity
Source: PLoS One. 2021 Mar 30;16(3):e0249339. doi: 10.1371/journal.pone.0249339 (PMC8009398; doi:10.1371/journal.pone.0249339)
Supplement: S1 Appendix — (DOCX) [file pone.0249339.s001.docx]

# S1 Appendix. Experimental instruction for the belief elicitation task

**Instruction**

On the next page, you will find the answer sheet to the same 12 games used in Part 1 of this experiment.

In this part, however, we would like you to:

1. indicate your guess about what strategy the other player chose in the first part of the experiment and;
2. assign a number *p* between 0 (absolutely uncertain) and 100 (absolutely certain) that best describes how certain you are about your guess.

**Payoff**

As in the first part of the experiment, one of the 12 games will be randomly drawn to determine your payoff.

Your payoff will be determined as follows:

| **Case 1:** If your guess about the other player’s strategic choice is correct, your payoff will be determined by the following formula: | |  | **Example** for the possible payoffs, if the guess is correct, as a function of the different values of p: | |
| --- | --- | --- | --- | --- |
| $200\cdot\left[ 1-\left( 1-\frac{p}{100} \right)^{2} \right]$ | p = degree of certainty |  | p = 0 🡪 0 Points | p = 60 🡪 168 Points |
|  |  |  | p = 10 🡪 38 Points | p = 70 🡪 182 Points |
|  |  |  | p = 20 🡪 72 Points | p = 80 🡪 192 Points |
|  |  |  | p = 30 🡪 102 Points | p = 90 🡪 198 Points |
|  |  |  | p = 40 🡪 128 Points | p = 100 🡪 200 Points |
|  |  |  | p = 50 🡪 150 Points |  |
|  |  |  |  |  |
| **Case 2:** If your guess about the other player’s strategic choice is wrong, your payoff will be determined by the following formula: | |  | **Example** for the possible payoffs if the guess is wrong, as a function of the different values of p: | |
| $200\cdot\left[ 1-\left( \frac{p}{100} \right)^{2} \right]$ | p = degree of certainty |  | p = 0 🡪 200 Points | p = 60 🡪 128 Points |
|  |  |  | p = 10 🡪 198 Points | p = 70 🡪 102 Points |
|  |  |  | p = 20 🡪 192 Points | p = 80 🡪 72 Points |
|  |  |  | p = 30 🡪 182 Points | p = 90 🡪 38 Points |
|  |  |  | p = 40 🡪 168 Points | p = 100 🡪 0 Points |
|  |  |  | p = 50 🡪 150 Points |  |

**In each game, please indicate which strategy you think the other player chose and indicate how confident (0-100) you are of your guess.**

| ***Game 1*** |  |  |  | Other player’s choice | | |  |
| --- | --- | --- | --- | --- | --- | --- | --- |
|  |  |  |  | Strategy A | Strategy B | |  |
|  |  | Your choice | Strategy A | 0 / 0 | 200 / 50 | |  |
|  |  |  | Strategy B | 50 / 200 | 60 / 60 | |  |
|  |  |  |  | **Strategy A** | **Strategy B** | |  |
| **Which strategy do you think the other player chose?** | | | | **□** | **□** | |  |
| **How confident (0-100) are you of your guess?**  Please use a number between 0 (absolutely uncertain) and 100 (absolutely certain). | | | | | |  |  |
