# Supplementary material for: Acute tryptophan depletion in healthy subjects increases preferences for negative reciprocity
Source: PLoS One. 2021 Mar 30;16(3):e0249339. doi: 10.1371/journal.pone.0249339 (PMC8009398; doi:10.1371/journal.pone.0249339)
Supplement: S1 Table — (DOCX) [file pone.0249339.s002.docx]

**S1 Table. Lottery choice task.**

| Lottery | Lottery A | Lottery B | A | B | Expected value differences* |
| --- | --- | --- | --- | --- | --- |
| 1 | [0, 0%; 200, 100%] | [50, 0%; 120, 100%] |  |  | 80 |
| 2 | [0, 10%; 200, 90%] | [50, 10%; 120, 90%] |  |  | 67 |
| 3 | [0, 20%; 200, 80%] | [50, 20%; 120, 80%] |  |  | 54 |
| 4 | [0, 30%; 200, 70%] | [50, 30%; 120, 70%] |  |  | 41 |
| 5 | [0, 40%; 200, 60%] | [50, 40%; 120, 60%] |  |  | 28 |
| 6 | [0, 50%; 200, 50%] | [50, 50%; 120, 50%] |  |  | 15 |
| 7 | [0, 60%; 200, 40%] | [50, 60%; 120, 40%] |  |  | 2 |
| 8 | [0, 70%; 200, 30%] | [50, 70%; 120, 30%] |  |  | -11 |
| 9 | [0, 80%; 200, 20%] | [50, 80%; 120, 20%] |  |  | -24 |
| 10 | [0, 90%; 200, 10%] | [50, 90%; 120, 10%] |  |  | -37 |
| 11 | [0, 100%; 200, 0%] | [50, 100%; 120, 0%] |  |  | -50 |

* Expected value differences = expected value lottery A – expected value lottery B. Participants did not see this column.
