# Supplementary material for: Acute tryptophan depletion in healthy subjects increases preferences for negative reciprocity
Source: PLoS One. 2021 Mar 30;16(3):e0249339. doi: 10.1371/journal.pone.0249339 (PMC8009398; doi:10.1371/journal.pone.0249339)
Supplement: S2 Table — (DOCX) [file pone.0249339.s003.docx]

**S2 Table. Robustness checks on the GEE regression testing the interaction effect of treatment and beliefs on strategy choices (i.e., Model 2).**

|  |  | GEE linear | |  |  | Mixed-effect logistic | |  |
| --- | --- | --- | --- | --- | --- | --- | --- | --- |
|  |  | Coef.  (SE) | z-value  (p-Value) |  |  | Coef.  (SE) | z-value  (p-Value) |  |
| ATD |  | - .10  (.09) | -1.12  (.264) |  |  | -2.49  (1.52) | -1.64  (.101) |  |
| Belief |  | .08  (.10) | .82  (.411) |  |  | -1.93  (2.35) | -.82  (.412) |  |
| ATD × Belief |  | .44  (.12) | 3.65  (<.001) |  |  | 8.36  (3.07) | 2.72  (.007) |  |
| Incentive DD |  | -.00  (.00) | -8.66  (<.001) |  |  | -.05  (.012) | -4.17  (<.001) |  |
| Risk attitude |  | .03  (.02) | 1.18  (.239) |  |  | .62  (.37) | 1.69  (.092) |  |
| Constant |  | .63  (.16) | 3.97  (<.001) |  |  | .92  (1.97) | .46  (.643) |  |
| N = |  | 47*12 |  |  |  |  |  |  |
| Wald χ² |  | 203.65  (<.001) |  |  |  |  | 29.59  (<.001) |  |
