# Supplementary material for: Acute tryptophan depletion in healthy subjects increases preferences for negative reciprocity
Source: PLoS One. 2021 Mar 30;16(3):e0249339. doi: 10.1371/journal.pone.0249339 (PMC8009398; doi:10.1371/journal.pone.0249339)
Supplement: S3 Table — (DOCX) [file pone.0249339.s004.docx]

**S3 Table. Strategy choices and beliefs over the incentive levels for mutual cooperation.**

| **Incentive DD** | **60** | **70** | **80** | **90** | **100** | **110** | **120** | **130** | **140** | **150** | **160** | **170** |
| --- | --- | --- | --- | --- | --- | --- | --- | --- | --- | --- | --- | --- |
| Hawks ATD  (SE) | 18/25  (.09) | 18/25  (.09) | 18/25  (.09) | 17/25  (.09) | 12/25  (.10) | 8/25  (.09) | 8/25  (.09) | 5/25  (.08) | 5/25  (.08) | 3/25  (.06) | 3/25  (.06) | 3/25  (.06) |
| Hawks Placebo  (SE) | 12/24  (.10) | 11/24  (.10) | 13/24  (.10) | 13/24  (.10) | 10/24  (.10) | 6/24  (.09) | 5/24  (.08) | 6/24  (.09) | 7/24  (.09) | 4/24  (.08) | 4/24  (.08) | 4/24  (.08) |
| Ø Belief ATD  (SE) | .578  (.08) | .518  (.08) | .500  (.07) | .486  (.07) | .398  (.07) | .347  (.06) | .282  (.06) | .256  (.06) | .242  (.06) | .212  (.05) | .191  (.05) | .172  (.05) |
| Ø Belief Placebo  (SE) | .510  (.07) | .499  (.07) | .464  (.07) | .437  (.07) | .371  (.06) | .293  (.05) | .262  (.05) | .226  (.04) | .190  (.04) | .179  (.04) | .155  (.04) | .140  (.04) |
